# Evaluating the spatial pattern of water quality of the Nile River, Egypt, through multivariate analysis of chemical and biological indicators

**DOI:** 10.1038/s41598-025-89982-2

**Published:** 2025-03-04

**Authors:** Mahmoud H. Hegab, Seliem M. El Sayed, Nasr M. Ahmed, Eman I. Abdel-Aal, Doaa A. Kassem, Khadiga M. Gaber, Amany M. Haroon, Soad S. Abdel Gawad, Mohamed E. Goher, Abd-Ellatif M. Hussian

**Affiliations:** 1 National Institute of Oceanography and Fisheries (NIOF), Cairo, 11694 Egypt; 2 Department of Biology, Faculty of Education, Matrouh University, Marsa Matrouh, 51511 Egypt

**Keywords:** The Nile, Physicochemical characteristics, River biota, Discriminant analysis, Water quality, Ecology, Environmental sciences, Limnology

## Abstract

The Nile River, known as the "giver of life," serves as Egypt’s main source of fresh water. A total of 28 sites along the Nile River were selected during the winter and summer of 2022 to illustrate spatial–temporal variation and indicate potential sources of pollution. The study showed that all chemical and physical parameters are within permissible limits. Based on the abiotic parameters, discriminant analysis (DA) classified the collected Nile sites into three groups (A, B, and C). Group A included the southern sites characterized by high transparency and low levels of electrical conductivity (EC), pH, dissolved oxygen (DO), biological oxygen demand (BOD), and chemical oxygen demand (COD). Group B included the middle sites and was characterized by the relatively high means of EC, total dissolved solid (TDS), pH, DO, BOD, and COD. Group C included the northern sites, which are characterized by low transparency and the highest value of nutrients and EC. A total of 113 phytoplankton species belonging to seven phyla were recorded, while a total of 52 zooplankton species were recorded. The lowest abundance and diversity of phytoplankton and zooplankton were detected at the southern sites, which increased gradually to attain the highest density and diversity in the northern sites. The submerged macrophyte species were recorded in the study area with low species diversity, and *Myriophyllum spicatum* was the dominant one. 98 epiphytic diatoms and 30 species of macroinvertebrates attached to macrophytes were recorded. The blood parameters of *Oreochromis niloticus* were within normal limits except for those collected from the Greater Cairo governorate sites in the north. The study showed an improvement in Nile water quality due to increased water levels and water discharged into the Nile.

**Supplementary Information:**

The online version contains supplementary material available at 10.1038/s41598-025-89982-2.

## Introduction

Throughout known Egyptian history, the Nile had dominating influences on the economy, culture, public health, social life, and political aspects^1^. The Nile River is the longest in the world and extends for approximately 6853 kms from its furthest source in the East African plateau (from the source of the Nyiragongo River in Rwanda and the Ruvubu River in Burundi) to the shores of the Mediterranean Sea, thus crossing approximately half of the African continent. The Nile drainage basin, with an area of approximately 325 × 105 km^2^, occupies one-tenth of the area of the African continent and is unique among the world’s rivers as it includes three climatic zones: tropical, subtropical, and arid desert. The Nile Basin extends over eleven riparian countries; Burundi, Rwanda, Tanzania, Zaire, Uganda, Kenya, the Central African Republic, Ethiopia, Sudan, and Egypt^2^. In Egypt, The Nile River extends from Aswan (below the High Dam) to Cairo with a length of 950 km. After passing through Cairo, the Nile divided into two main branches, Rosetta and Damietta^2,3^. Although the Nile River is the only source of fresh water in Egypt, it faces many challenges. One of the important issues is the increasing environmental pressure due to pollution from various sources, including industrial wastewater, agricultural runoff, and sewage discharge. The discharge of untreated wastewater significantly alters the physical, chemical, and biological properties of the river^4^. About 124 drains are the wastewater sources of pollutants in the Nile from Aswan to Cairo. A large part of this pollution stems from agricultural drainage (67 agricultural drains), which carries untreated pollutants directly into the river^5–7^. Also, the minerals sector contributes a significant amount of waste to the river, exacerbating the pollution problem^6,8^. The consequences of this pollution are severe, posing risks to the health of the river ecosystem and potentially affecting human health. Furthermore, the recent construction of the Ethiopian dam has exacerbated these issues, creating additional environmental and health concerns^9^. Wahaab and Badawy^10^ and Abdel-Satar^11^ stated that the Nile River receives large quantities of untreated wastewater rich in organic matter, which directly affects the banks of the river, while the midstream water is slightly affected. However, we can suggest that the Nile River has a great capacity for self-purification. The high level of pollution resulting from the decline in the Nile River water level has become a major problem facing Egypt, especially after the completion of the Ethiopian dam^12,13^. The water quality of the Nile is a major concern, driven by the expansion of industrial, agricultural, and recreational activities, coupled with an unregulated drainage system. As the Nile is Egypt’s primary source of drinking and irrigation water, continuous monitoring of water quality and environmental status is paramount^14^. Water quality can be assessed using physical and chemical properties, which reveal the characteristics of the water and the variables affecting its quality. However, incorporating biological properties into the assessment provides a more complete picture, reflecting the extent to which changes in a given body of water impact the organisms that inhabit it^15^. For example, phytoplankton and zooplankton have become good indicators commonly used to monitor environmental conditions^16^ and they respond quickly to any changes in water quality^17–20^. Moreover, aquatic organisms provide many ecological benefits and are essential in enhancing the diversity and function of aquatic systems^21^.The current study used chemical and biological indicators to evaluate surface water quality. Unlike previous studies of the Nile River, which focused on drainage areas and collected samples from limited distances before and after drainage (revealing significant pollution), the current study adopted a different approach. Sampling sites were selected based on geographical distances and represent all governorates along the Nile, rather than focusing solely on drainage sites. This strategy aims to determine the true environmental status of the Nile and assess the effectiveness of its self-purification processes.

## Material and methods

### Study area

The Nile River is the main water source for Egypt, and the traditional concern with securing sufficient water for Egypt’s survival and economic development cannot be overemphasized^22^. The Nile River enters Egypt from the southern border with Sudan. It flows through a narrow valley ranging from 2 to 20 km wide and extends for about 950 km from Aswan (below Lake Nasser) to the north of Cairo. About 25 km north of Cairo, the Nile River divides into two famous branches, the Rosetta and the Damietta, and the four main rayahs, which form a delta at its base on the Mediterranean Sea. Egypt’s great agricultural system connected to the Nile extends for approximately 31,000 km, containing main branches, canals, rayahs, and minor streams. According to Goher et al.^1^, the Nile along its extension in Egypt receives various wastes from more than 90 hotspots, including untreated domestic sewage, industrial point and non-point sources, and agricultural drains^1^.

### Sampling and analysis

#### Abiotic parameters

**Fig. 1:**
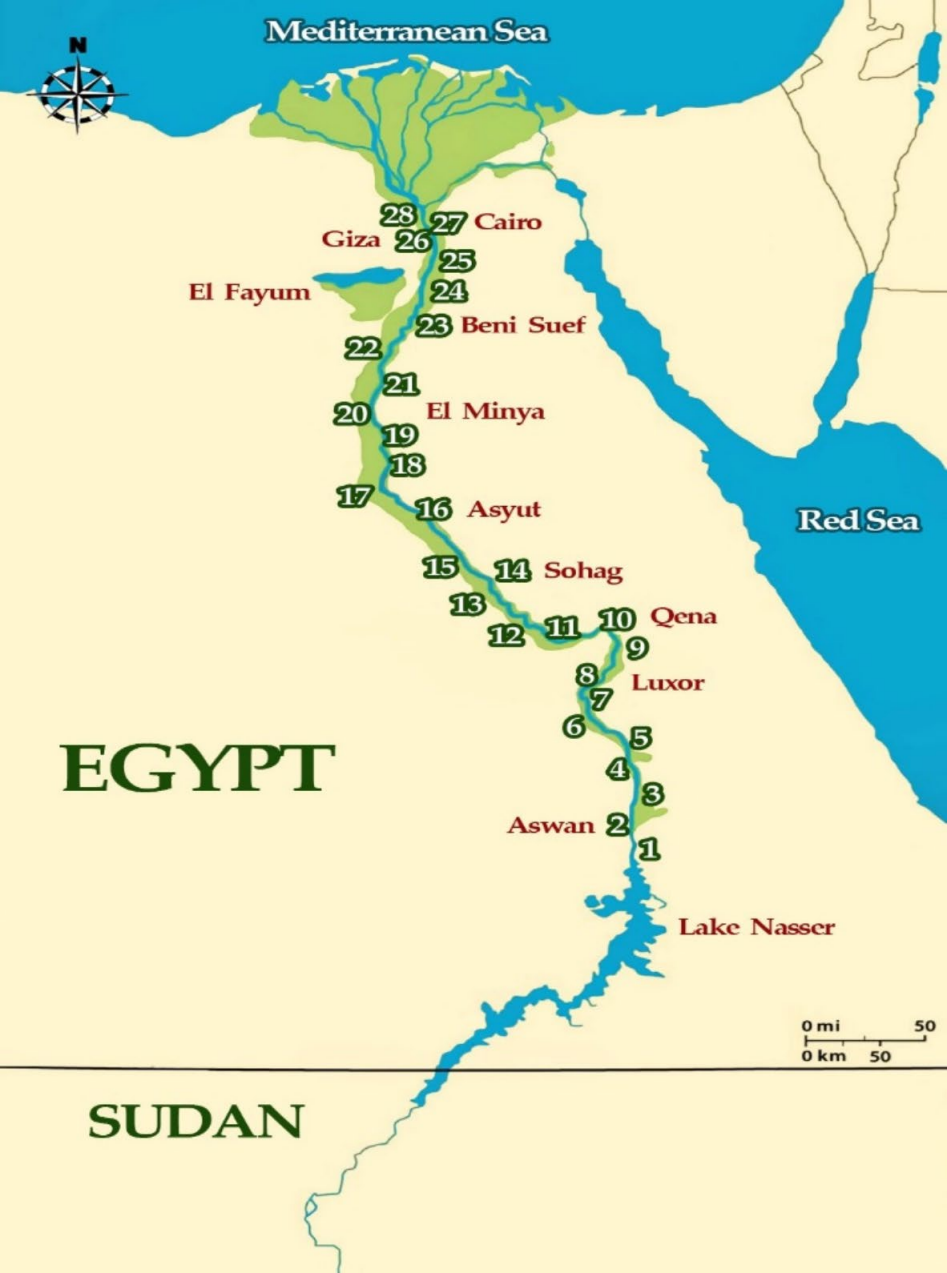
Map of Nile illustrates the sampling sites.

Subsurface water samples were collected using a 2L Ruttner water sampler (KC Denmark A/S) at 28 sites along the Nile from Aswan to Cairo (Figure 1 and Table S1 -in the Supplementary data-). A total of 56 duplicate water samples were collected in the winter and summer of 2022. For chemical analysis, the water samples were kept in polyvinyl chloride bottles and stored in ice boxes. Special stoppered bottles of 300 cm^3^ capacity were used to collect the water samples of dissolved oxygen (DO) and biochemical oxygen demand (BOD). The samples of DO were fixed directly using 1 ml of manganous sulfate (40%) and 1 ml alkaline potassium iodide solution. In the meantime, the BOD bottle samples were covered with aluminum foil to reflect light.

The standard methods in APHA^23^ were used to determine the physico-chemical features of water samples. In situ, pH, EC, and water temperature were measured using a multiparameter (Hanna HI9829, Woonsocket, RI, USA). Total Dissolved Solids (TDS) and total suspended solids (TSS) were measured by evaporation at 180 °C. DO and BOD were measured using the modified Winkler method, while the dichromate method was used to determine the chemical oxygen demand (COD). A double-beam Jenway 6800 spectrophotometer was used to determine the concentrations of the nutrient salts by the phenate method for nitrogen–ammonium (N–NH_4_), the colorimetric method with the formation of a reddish-purple azo dye for nitrogen–nitrite (N–NO_2_) and nitrogen–nitrate (N–NO_3_ after cadmium reduction), the ascorbic acid molybdate method for orthophosphates (P–PO_4_), and the molybdosilicate method for silicates (SiO_4_). Total phosphorus (TP) was determined as PO_4_ after simultaneous persulfate digestion^24^. 6 readings for each analysis (3 of each duplicate sample) with relative standard deviations less than 10% were used to determine the precision of the water quality analysis.

#### Biotic parameters

The phytoplankton was sampled, preserved, and subjected to examinations. For quantitative and qualitative analysis of phytoplankton, 500 ml of water was collected from each site and immediately preserved using Lugol’s iodine. The samples were transferred into a glass cylinder and left to settle for 5 days. About 90% of the supernatant was siphoned off using plastic tubes covered with a plankton net (5μ) and adjusted to a fixed volume. Lugol-preserved subsamples were prepared for species identification and enumeration using an inverted microscope (ZEISS IM 4738, with a magnification of 400 × and 1000 × (with oil immersion). Each sample was examined and counted using the drop method^23^. The primary references used in phytoplankton identification^25–29^ as mentioned by Abdel-Aal et al^30^. Zooplankton samples for quantitative analysis were collected from the surface water of each surveyed site. Fifty liters of water were filtered through a 55 µm mesh plankton net (25 cm diameter, 80 cm length), then preserved with 4% formaldehyde. Laboratory analysis involved examining three 1 ml subsamples of homogenised zooplankton using the Sedgwick Rafter plankton counting cell under a binocular microscope (100-400X magnification) and counting to determine population density (individuals/m^3^) using a method adapted from APHA^23^. Species identification followed several key references^31–35^.

The submerged macrophytes were carefully harvested by carefully removing them from the substrate and put in plastic bags in a darkened cooler. In the laboratory they were separated into different taxa, identified based on Boulos^36^, and the species presence was expressed as percent of sites with taxa. Epiphytic diatom communities were collected by cutting three undisturbed bundles of submerged macrophytes using scissors into a bucket filled with filtered river water and then macrophytes were shacked vigorously in a plastic bottle until all attached communities were de-attached. Preserved samples were left to air-dry, then homogenized. In order to identify diatoms, it was necessary to remove organic material that obscures the delicate sculpturing on the silica valve. 0.5–2 g of homogenized samples were transferred into a tightly closed 100 ml tephlon bottles. The samples underwent acid treatment using 5 ml sulphuric acid (96%) and 10 ml nitric acid (96%) added to the samples. The samples were heated until all organic matter had been oxidized. The samples were washed and concentrated to a fixed volume (50 ml). Cleaning and digestion of the diatom samples were occurred according to ANS^37^. Preparation of permanent slides using a high refractive index medium (Naphrax) was performed (ANS^37^). Diatom identification and counting were achieved along random transect using a light microscope (Zeiss, Model Axiovert 25C) at a 40 × magnification and 100 × if needed. From each permanent slide, at least 400 valves were enumerated on random transects, and the count data were converted to percentage relative abundance of the total count. Identifications were made at the species and variety level according to Krammer and Lange-Bertalot; Taylor et al.^38–41^. Taxonomic status was updated according to the AlgaeBase web site^42^. For attached macroinvertebrates, macrophytes were put in filter papers to remove water and weighed as a fresh weight. In the laboratory, samples (washing products) were washed again in net 500 μm. Macroinvertebrates were separated under the microscope into groups, and they were identified as different taxa and species. Each species was counted, and the population density was estimated and expressed as a number of organisms/kg fresh weight of macrophytes.

The samples of Nile tilapia were collected in the winter and summer seasons from eight governances (Aswan, Luxor, Qena, Sohag, Assiut, El-Minya, Beni-surf, and Greater Cairo) along the River Nile from the High Dam to the Delta Barrages. Blood samples were collected by severance of the caudal peduncle of fish. Each sample was divided into two parts; the first one was mixed with an anticoagulant (EDTA) to prevent clotting to determine the haemoglobin, according to Blaxhall and Daisley^43^. The second part was left to clotting, then serum was taken and centrifuged at 3000 rpm for 10 min. The supernatant was collected using a micropipette model (Lab Systems K 33071) for determination of serum glucose concentration, which was done using the method of Trinder^44^; total protein^45^; creatinine^46^; uric acid and urea^47^; aspartate aminotransferase (AST); and alanine aminotransferase (ALT) by Reitman and Frankel^48^ were determined calorimetrically using commercial kits (Spectrum, Egypt).

#### Data analysis

The XLSTAT 2019 program is used to express the descriptive statistics, including the mean, standard deviation, minimum, and maximum values. The associations between the studied abiotic variables were examined using the Pearson correlation coefficient using Excel-Stat 2019 software. The obtained results were analyzed using the Excel-Stat 2019 software for determination of the spatial and temporal variations by analysis of variance using the one-way ANOVA test (alpha = 0.05) which was also used for determination of the probability values (*P-values*); significance levels of tests were taken as significant when *P* ≤ 0.05 and highly significant when *P* ≤ 0.01. In addition, Discriminant analysis grouped similar sites along the River Nile from Aswan to Cairo based on chemical and biological data, in addition to fish blood data using XLSTAT 2019 software. The canonical corresponding analysis (CCA) was applied to show the relationship between the most abundant phytoplankton, zooplankton, epiphytic diatoms, epiphytic macroinvertebrates species, and the main chemical parameters using XLSTAT 2019 software. Also, after the DCA analysis, CCA was used to show the relation between water quality parameters and fish blood parameters. According to the data of DCA, where the gradient length > 4 of standard division, principal component analysis (PCA) was used to show the relationship between the chemical parameters of water quality using XLSTAT 2019 software.

## Results

### Physico-chemical characteristics

**Fig. 2:**
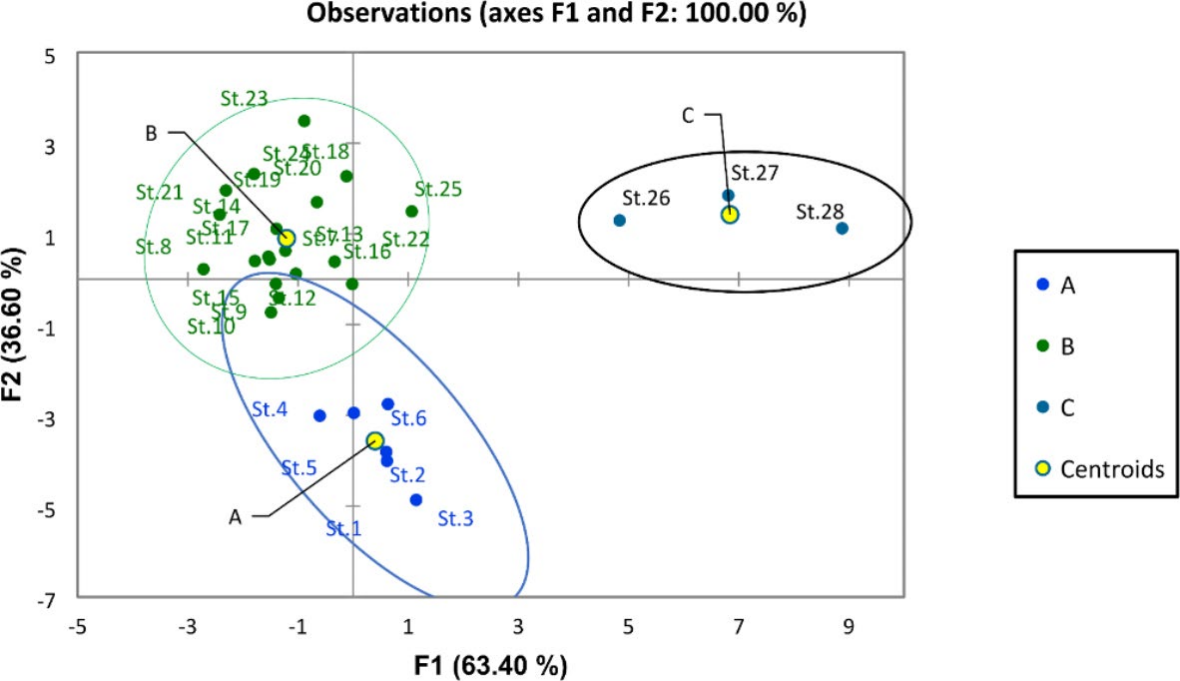
Discriminant analysis clustered the river Nile sites into three groups according to the water quality parameters.

**Fig. 3:**
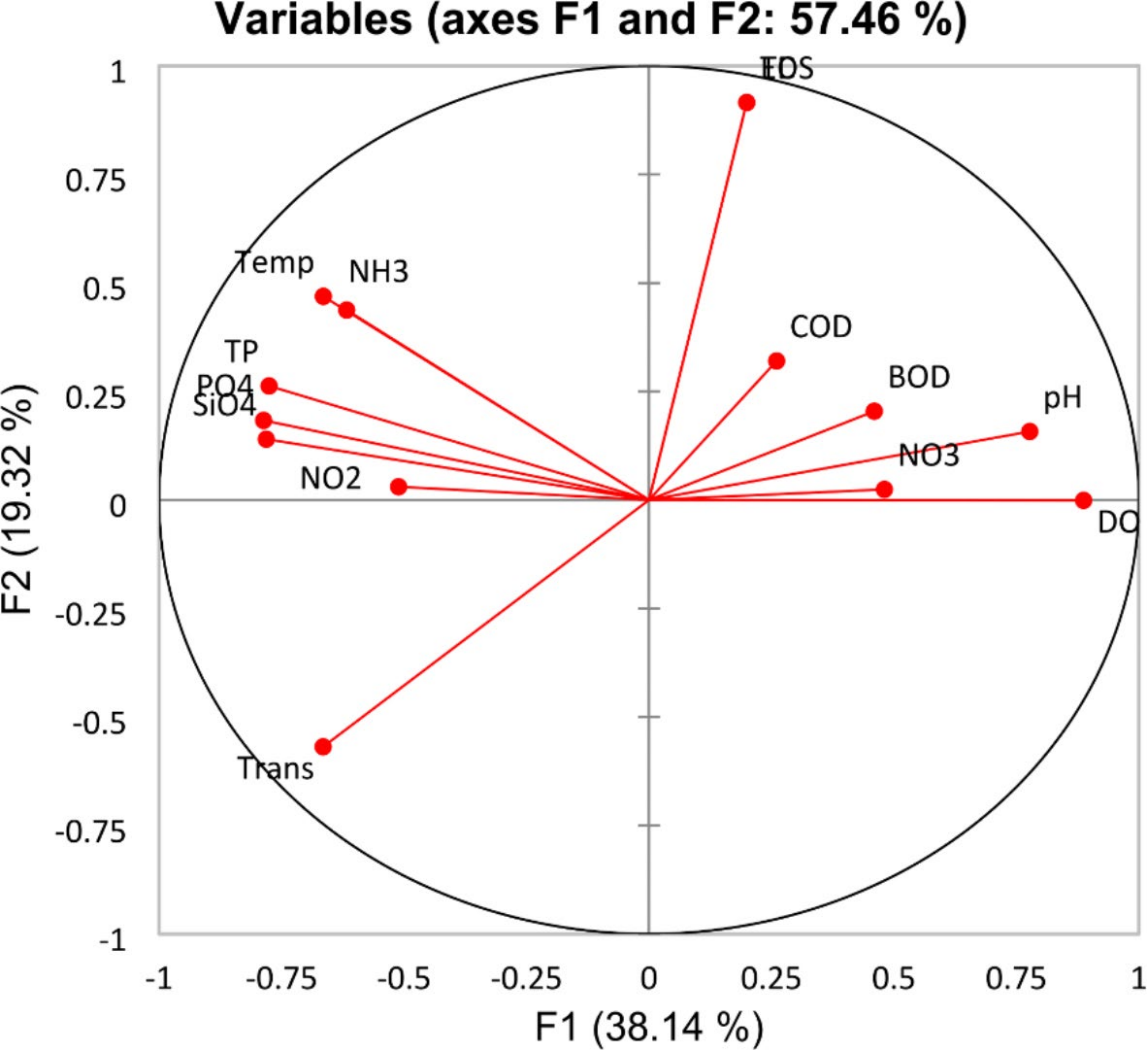
PCA analysis among the water quality parameters.

Physicochemical features of the Nile water including hydro-chemical properties and nutrient salts have been examined with discriminant and PCA analysis. The discriminant analysis based on water quality parameters assembled the different sites of the Nile into three groups (A, B, and C) (Fig. 2). Group A included the southern sites (St. 1, 2, 3, 4, 5, and 6) from Aswan Province and the first station from Luxor. This group was characterized by high transparency, low EC, TDS, pH, DO, BOD, and COD (Fig. 3). Groups A and B overlapped. Group B included the middle sites (from Luxor to Giza Province (St. 7 to Station 25). It was characterized by the relatively high means of EC, TDS, pH, DO, BOD, and COD. And relatively low value of transparency, On the other hand, group C included the northern sites in Cairo and Qalyubia provinces (St. 26, 27, and 28). This group is characterized by the lowest values of transparency and the highest values of ammonia, nitrite, nitrate, EC, and TDS.

Obtained data (Table 1) indicate that the temperature of the Nile water during the study period was within the best range for fish and aquatic organisms and ranged between 15.9 and 29.95 °C with a highly significant difference between seasons (*p* < 0.01) (Table 1). Transparency decreases gradually from south to north, recording the minimum value of 80 cm at sites 23 and 24 in winter and the maximum of 500 cm at site 1. EC and TDS showed an opposite distribution pattern to transparency and increased gradually from south to north and varied between 260–427 μS/cm and 169–277.55 mg/l, respectively, with highly significant differences between sites (*p* < 0.01). In the same manner, the pH on the alkaline side increased from south to north and ranged between 7.51 and 8.55, with a significant difference between seasons and sites (*p* < 0.01). DO results showed a high significant difference between seasons and sites (*p* < 0.01), where a low DO level was recorded in the south region, especially in the summer seasons that reacted to the lowest value of 2.47 mg/l at site 1, while the maximum one of 10.4 m/L was found at site 8 in winter. In general, BOD and COD recorded low-moderate levels during the study period and ranged between 0.84–5.9 and 1.75–10.37 mg/l, respectively. ANOVA results show that NH_4_ has a high significant difference between site and seasons (*p* < 0.01), while NO_3_, PO_4_, TP, and SiO_4_ showed high significant differences between seasons (*p* < 0.01). Nutrient salts, NO_2_, NO_3_, NH_4_, PO_4_, TP, and SiO_4_, fluctuated in the following ranges: ND–18.37 µg/l, 11.52–280.75 µg/l, 35.7–341.7 µg/l, 1.1–33.11 µg/l, 7.49–60.8 µg/l, and 0.34–14.59 mg/l, respectively.

**Table 1:** The physicochemical characteristics of water in River Nile in winter and summer, 2022.

Parameters	Winter				Summer			
	Min	Max	Mean	SD	Min	Max	Mean	SD
Temp 0C	15.09	19.99	17.49	1.21	23.42	29.95	26.98	2.03
Trans cm	80	500	161.43	94.09	110	480	235.36	110.9
EC µS/cm	260	427	309.57	43.81	263	395	316.76	37.12
TSS mg/l	5	26	15.39	5.52	6	22	19.07	5.23
TDS mg/l	169	277.55	201.22	28.48	170.95	256.75	205.89	24.13
pH	7.63	8.55	8.18	0.21	7.51	8.29	7.85	0.23
DO mg/l	5.86	10.4	7.72	0.86	2.47	7.52	5.52	1.21
BOD mg/l	1.25	5.9	3.14	1.16	0.84	5.4	2.77	1.21
COD mg/l	1.98	9.32	4.89	1.85	1.75	10.37	5.36	2.28
NH_4_ µg/l	35.7	256.1	100.46	58.18	115.6	341.7	190.83	64.11
NO_2_ µg/l	0.76	18.37	3.89	3.67	ND*	14.84	5.44	3.19
NO_3_ µg/l	32.98	280.75	105.73	65.55	11.52	117.36	33.99	19.47
PO_4_ µg/l	1.1	12.1	6.33	2.7	2.14	33.11	17.59	9.63
TP µg/l	7.49	20.12	13.61	3.6	12.2	60.08	33.53	15.52
SiO_4_ mg/l	0.34	4.19	1.66	1.21	2.2	14.59	7.54	3.55

*ND: not detected.

PCA analysis (Fig. 3) illustrates the relation between the chemical parameters in the Nile water at the different sites. It has been noted that the temperature is positively correlated with most nutrient salts (except nitrate). In contrast, there was a significant negative relation between the temperature degree and the values of DO, pH, and nitrate. On the other hand DO is positively correlated with nitrate, BOD, COD, and pH. In contrast to temperature, DO is negatively correlated with most nutrient salts (NH_4_, NO_2_, PO_4_, TP, and SiO_4__._). On the other side, transparency is negatively correlated with EC, TDS, pH, and DO. These findings are confirmed by the Pearson correlation values between the different parameters (n = 56 and *p* < 0.01), such as temperature/NH_4_ (*r* = 0.49), temperature/PO_4_ (*r* = 0.53), temperature/TP (*r* = 0.59), temperature/SiO_4_ (*r* = 0.67), temperature/pH (*r* = − 0.43), temperature/DO (*r* = − 0.58), temperature/NO_3_ (*r* = − 0.61), DO/NH_4_ (r = − 0.67), DO/NO_2_ (*r* = − 0.53), DO/PO_4_ (*r* = − 0.55), DO/TP (*r* = − 0.53), and DO/SiO_4_ (*r* = − 0.53).

### Phytoplankton assemblages

The phytoplankton communities in the Nile River comprised 113 species belonging to seven phyla. In winter, 18 Cyanobacteria, 53 Chlorophyta, 31 Heterokontophyta, 3 Cryptophyta, 4 Miozoa, 3 Euglenophyta, and 1 Ochrophyta were identified, while in summer, 16 Cyanobacteria, 58 Chlorophyta, 31 Heterokontophyta, 2 Cryptophyta, 2 Miozoa, and 1 Euglenophyta were identified. The algal densities and species richness of the Nile show remarkable spatial and seasonal variations, with higher values downstream of the Nile. The range of species recorded was 13–50 species and 13–53 species in winter and summer, respectively. In winter, the total phytoplankton count ranged from 12.55 to 46.45 × 10^6^ cells/L, and in summer, from 8.74 to 41.15 × 10^6^ cells/L. The low recorded phytoplankton densities were in the southern stations from St. 2 to St. 12 (12.55–36.5 × 10^6^ cells/L; winter & 8.74–20.82 × 10^6^ cells/L; summer).

The cyanobacteria, diatoms, and green algae make up the greater part of the phytoplankton, with the remaining phyla contributing much less. Cyanobacteria were the dominant taxonomic group and accounted for 7–83.5% of total phytoplankton abundance in winter and from 26.5 to 78.6% in summer. Green algae made up an average of 23% in the winter and 30% in the summer. The phytoplankton standing crop was dominated by those species with wide-range survivals and not affected by the season (e.g., Cyanobacteria: *Chroococcus dispersus var. minor*, *Cylindrospermopsis raciborskii* (Wołoszyńska) Seenayya et Subba Raju, *Merismopedia punctata* Meyen, and *Pseudoanabaena* spp., green algae: *Ankistrodesmus falcatus* var. *acicularis* (A. Braun), *Botryococcus* spp., *Chlamydomonas globosa* J.W. Snow, *Chlorella vulgaris* Chodat, *Dictyosphaerium pulchellum* H.C. Wood, *Micractinium* *pussillum* Fresenius, and *Westella botryoides* (West) De Wildeman, whose shared percent varied between 5.5 and 31.7%. Meanwhile, in summer, the frequent species were *Actinastrum hantzschii* Lagerheim, *Ankistrodesmus falcatus* var. *acicularis* (A. Braun) G.S. West, *Botryococcus spp., and* *Chlamydomonas globosa.* J.W. Snow, *Choricystis chodatii* (Jaag) Fott, *Coelastrum cambricum* W. Archer, *Micractinium bornhemiense* (W. Conrad) Korshikov, *Micractinium pussillum* Fresenius, *Scenedesmus bijugatus* Kützing, *S.* *quadricauda* (Turpin) Brébisson, and *Stigocolonium* sp. Diatoms: *Aulacoseira granulata* (Ehrenberg) Simonsen, *Cyclotella meneghiniana* Kützing, *C. ocellata* Pantocsek, *Asterionella formosa* Hassall, *Staurosirella leptostauron* (Ehrenberg), and *Ulnaria ulna* (Nitzsch) Compère. The most notable finding was the presence of *Synechococcus* sp. in the stations (4–9) with a percent share of the total count of 1–48% in the summer season. Also, the pennate diatom *Navicula muralis* Grunow was present at St. 2 in the winter with 81% of the total phytoplankton abundance.

**Fig. 4:**
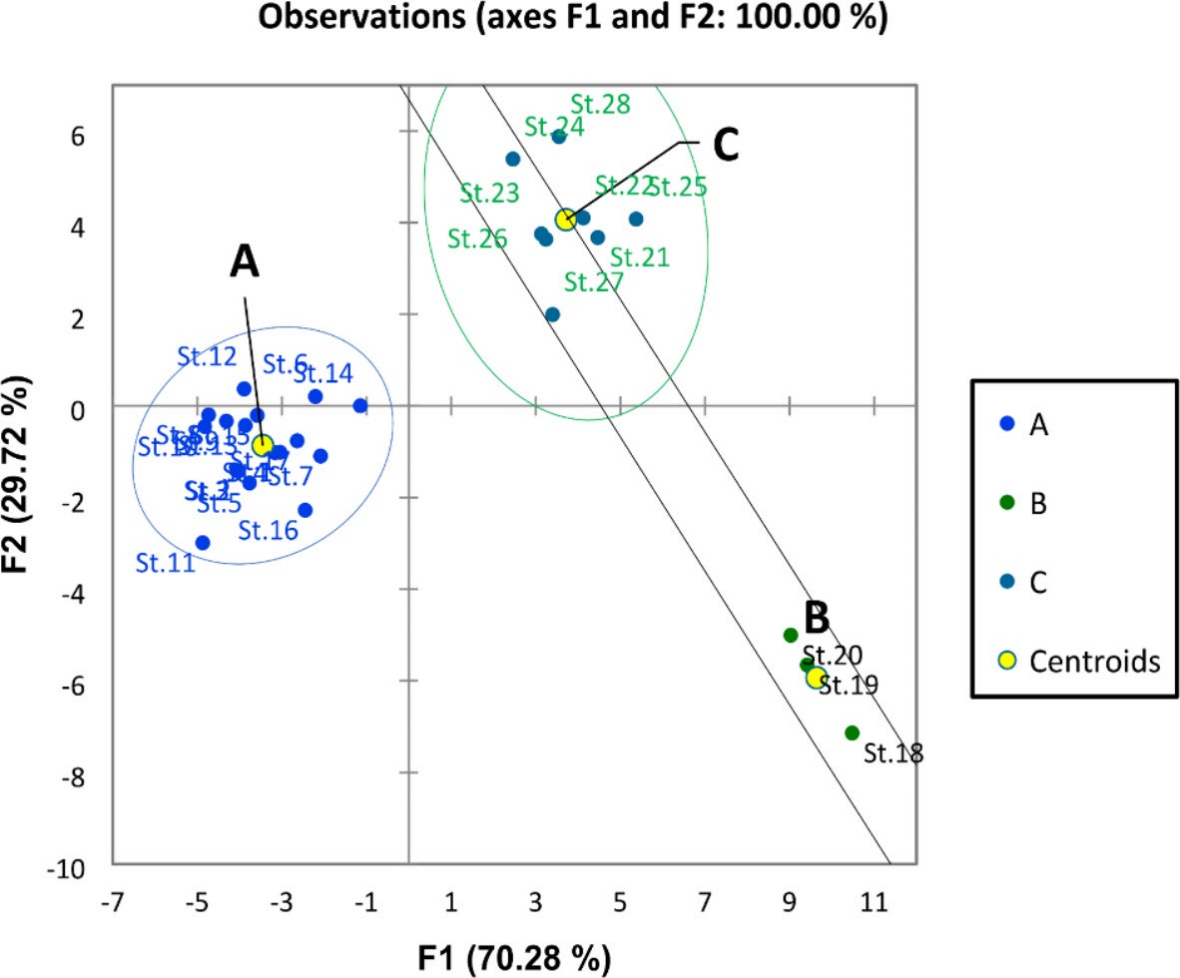
The discriminant analysis (DA) of the Nile sites depending on the common phytoplankton species in the Nile water.

**Fig. 5:**
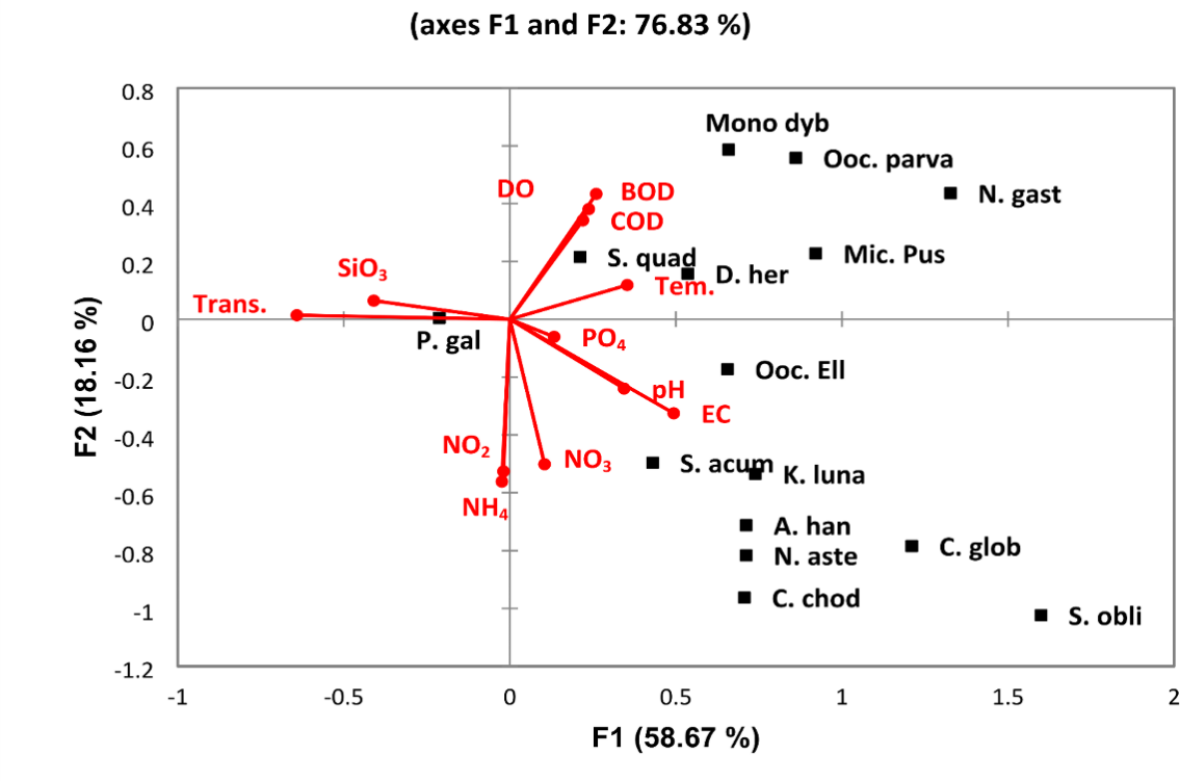
Canonical Correspondence Analysis (CCA) triplot diagram for the relations between the most common phytoplankton species (squares) and the physicochemical variables (lines). The used parameters are abbreviated as follows: temperature (Tem,), transparency (Trans.), pH, electrical conductivity (EC), dissolved oxygen (DO), biological oxygen demand (BOD), chemical oxygen demand (COD), nitrite (NO_2_), nitrate (NO_3_), ammonia (NH_4_), phosphorous (PO_4_), silicate SiO_4_, *Pseudanabaena galeata* (P. gal), *Actinastrum hantzschii* (A. han), *Chlamydomonas globosa* (C. glob*), Choricystis chodatii* (C. chod), *Dictiosphaerium ehrenbergianum* (D. her), *Kirchneriella lunaris* (K. luna), *Micractinium pusillum* (Mic. Pus), *Monoraphidium dybowskii* (Mono dyb*), Oocystis elliptica* (Ooc. Ell), *Oocystis parva* (Ooc. Parva), *Scendesmus obliquus* (S. obli), *Scendesmus acuminatus* (S. acum), *Scenedesmus quadricauda* (S. quad), and *Navicula gastrum* (N. gast).

The discriminant analysis (DA) for the common phytoplankton species in the Nile water differentiated the Nile River into three groups. The first group (A) was represented by the southern sites (St. 1–17). This group was characterized by the lowest phytoplankton abundance and diversity. The second group (B) included the middle sites (St. 18–20), while the third group included the sites from St. 21 to St. 28 in the north; this group is characterized by the highest phytoplankton density and diversity (Fig. 4). The CCA clarifies the relation between the phytoplankton species and the water variables (Fig. 5). A cumulative correlation effect of the physicochemical variables on the indicator green and diatom algae species was evident (Fig. 5). A remarkable correlation was obtained between DO, BOD, COD, and temperature with the green algae species *Dictiosphaerium ehrenbergianum, Monoraphidium dybowskii*, *Micractinium pusillum, Oocystis parva, Scenedesmus quadricauda,* and the diatom *Navicula gastrum.* Meanwhile, *Actinastrum hantzschii*, *Chlamydomonas globosa, Choricystis chodatii*, *Kirchneriella lunaris*, *Oocystis elliptica*, *Scendesmus obliquus,* and *Scendesmus acuminatus* were influenced by EC, pH, NO_2_, NO_3_, NH_4_, and phosphorus (Fig. 5). The cyanobacteria species *Pseudanabaena galeata* was separated from the other species and was positively correlated by the water transparency and silicate.

#### Zooplankton

**Fig. 6:**
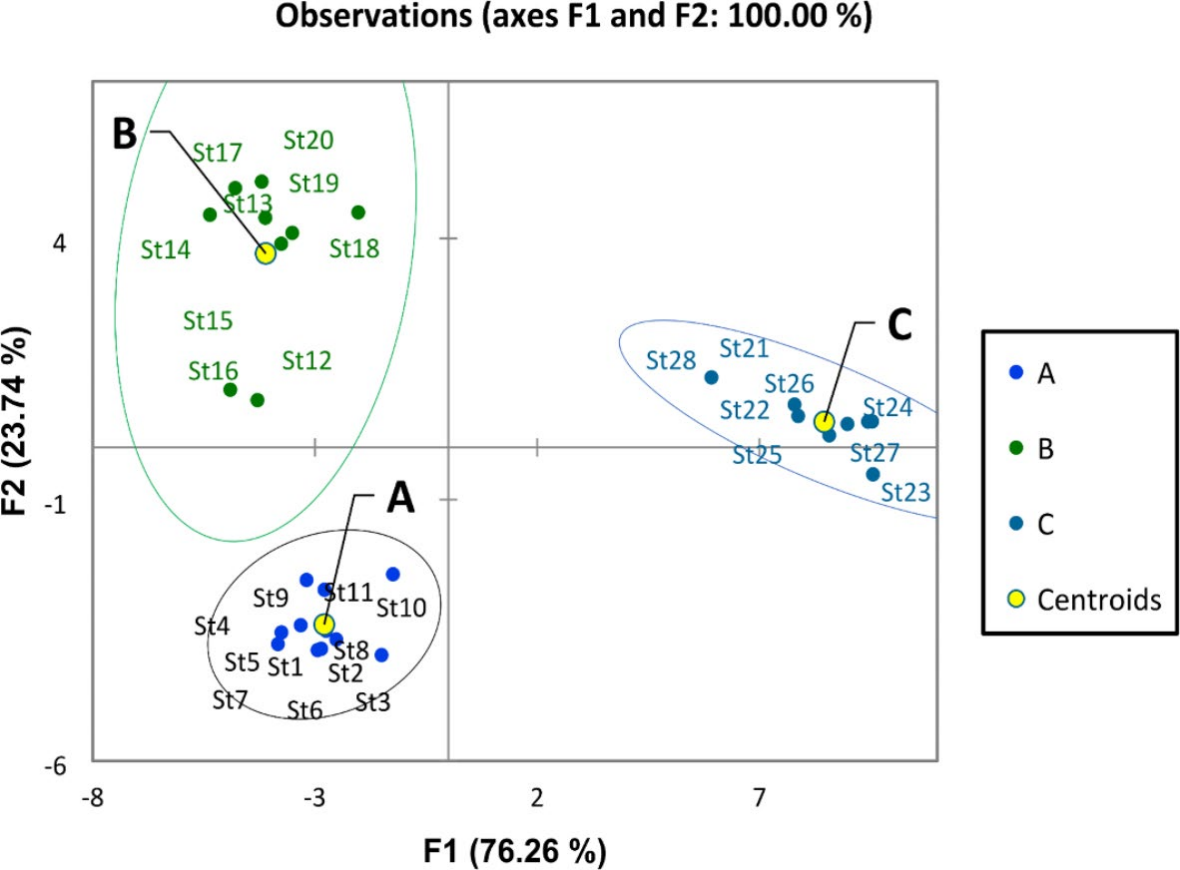
Discriminant analysis clustered the Nile River sites in three groups according to the distribution of zooplankton species in 2022.

**Fig. 7:**
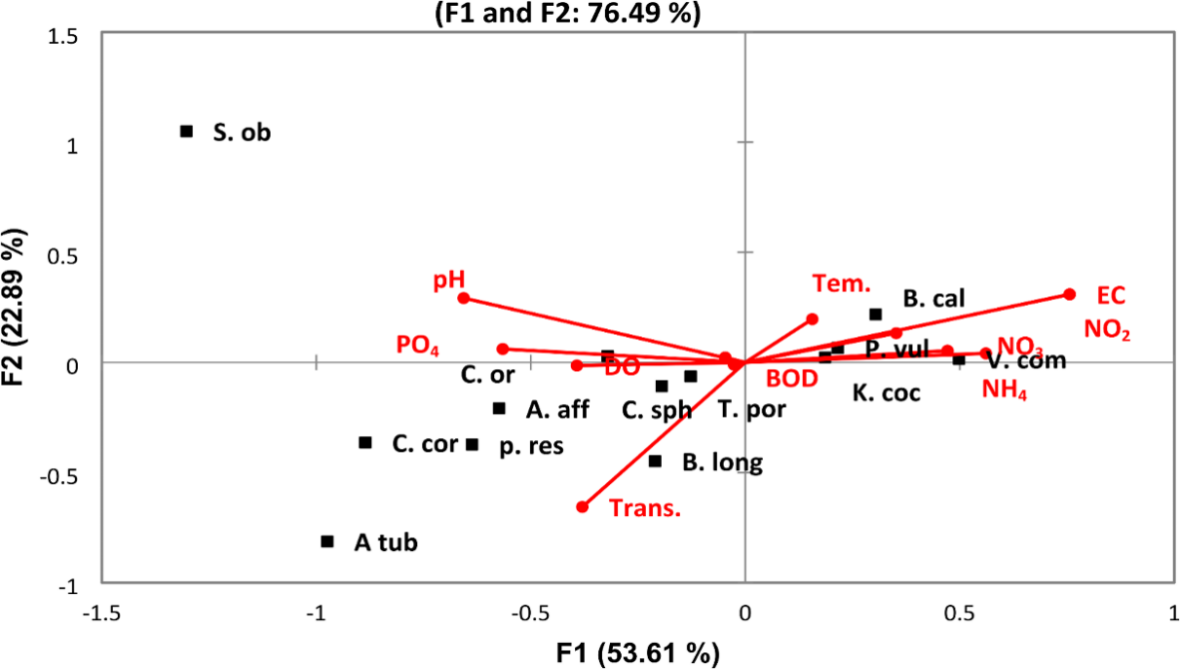
Canonical Correspondence Analysis (CCA) triplot diagram for the relations between the most common zooplankton species (squares) and the physicochemical variables (lines). The used parameters are abbreviated as follows: temperature (Tem,), transparency (Trans.), pH, electrical conductivity (EC), dissolved oxygen (DO), biological oxygen demand (BOD), chemical oxygen demand (COD), nitrite (NO_2_), nitrate (NO_3_), ammonia (NH_4_), phosphorous (PO_4_), silicate SiO_4_, *V. campanula* (*V. cam*), *Collotheca ornate* (C. or), *P. roseola* (p. res), *Trichocerca porcellus* (T. por), *B. calyciflorus* (B.s cal), *Synchaeta oblonga* (S. ob), *K. cochlearis* (K. coc), *P. vulgaris* (P. vul), *Ceriodaphnia cornuta* (C. cor), *B. longirostris* (B. long), *C. sphaericus (C. sph)* and *Alona affinis* (A. aff) in the River Nile (2022).

The density of zooplankton was higher in the winter season with an average of 1,485,000 Ind/m^3^ than in the summer (1,116,529 Ind/m^3^). Zooplankton was highly abundant downstream sites of the River Nile (Great Cairo) with a maximum density of 1,966,500 Ind/m^−3^ at St. 26 in winter seasons. A total of 52 zooplankton species were identified along the River Nile, including 41 species in winter and 42 species in summer. Rotifera was the dominant group, which was represented by 34 species. It formed 92.6 and 90.6% of the total zooplankton population in winter and summer, respectively. Among the rotifers, *Keratella cochlearis* was the dominant species (63.8% of total Rotifera) in winter, while *Brachionus calcyflouris* was the most dominant one (34.7%) in summer. Cladocera was the second predominant group of zooplankton (3.2% of total zooplankton). Among 9 cladocern species, *Bosmina longirostris* was the most dominant one and formed 52.5% and 40% of the total cladocera in summer and winter, respectively. Copepoda was represented by three adult species, in addition to their larval forms. The highest percentage (6% of total zooplankton) of Copepoda was detected in summer, while it decreased to 0.5% of total zooplankton in winter. The protozoa group disappeared during the summer, while 4.9% of the zooplankton was recorded in the winter, of which the dominant species was *Vorticella campanula*. Similar to water quality parameters and phytoplankton, the discriminant analysis (Fig. 6) divided the study area into three groups. The first group (A) includes sites from St. 1 to St. 11 (upstream), which was characterized by the lowest density, and the second group (B) from St. 12 to St. 20, which had a medium density of zooplankton. Zooplankton density was increased in the third group (C), which includes sites from St. 21 to St. 28 (downstream). Furthermore, the third group was characterized by the high abundance of *Keratella cochlearis, Brachionus calcyflouris, Philodina roseola, Polyarthra vulgaris, Collotheca ornate, Bosmina longirostris, Chydorus sphaericus,* and *Vorticella campanula.* The CCA analysis showed that NO_3_, NH_4_, BOD, COD, pH, and DO were the most abiotic factors affecting the most abundant species. These factors were positively correlated with *K. cochlearis, B. calcyflouris, P. roseola, P. vulgaris, C. ornate, B. longirostris, C. sphaericus,* and *V. campanula* (Fig. 7).

### Macrophytes

**Fig. 8:**
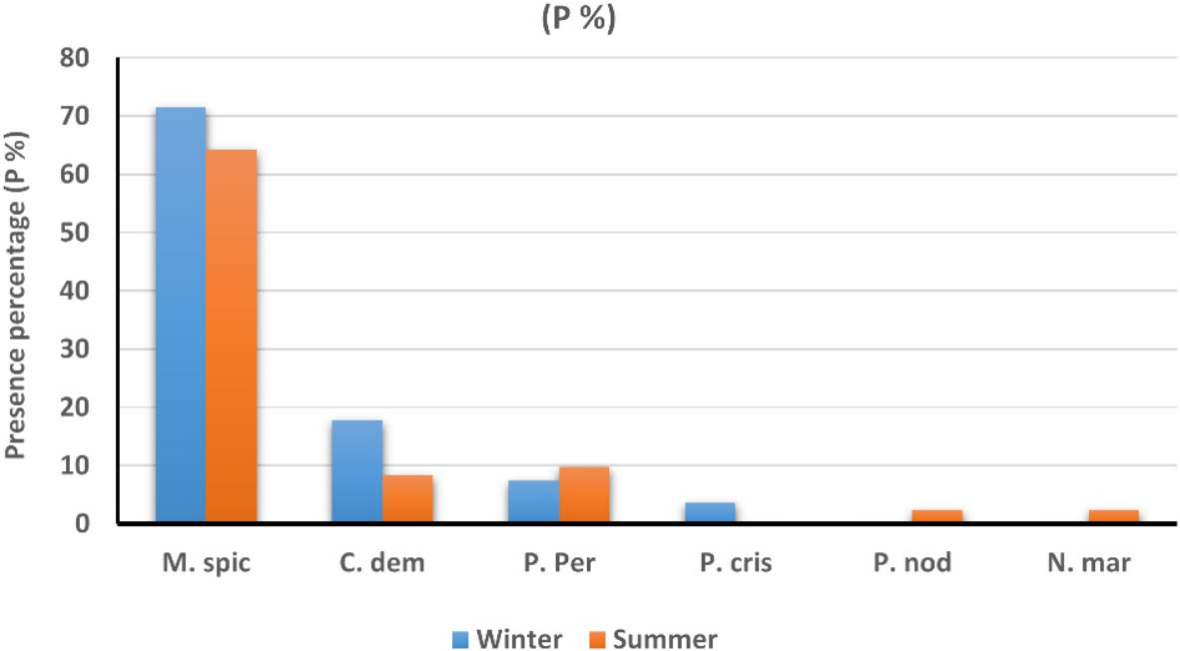
Presence percentage (P %) of each macrophyte species in relation to the total sampling sites of the surveyed area during winter and summer seasons; *Myriophyllum spicatum* = *M. spic, Ceratophyllum demersum* = *C. dem, Potamogeton perfoliatus* = *P. perf, Potamgeton crispus* = *P. cris, Potamogeton nodosus* = *P. nod* and* Najas marina* = *N. mar.*

Four submerged macrophyte species belonging to three genera and three families, including *Myriophyllum spicatum (*Haloragaceae), *Ceratophyllum demersum* (Ceratophyllaceae), *Potamogeton perfoliatus, and Potamogeton crispus* (Potamogetonaceae), were recorded during the winter season. *Myriophyllum spicatum* was the most frequent species observed at 71.5% of total macrophytes sampling sites (Fig. 8). There were geographical differences in species distribution, where *Myriophyllum spicatum* was found alone in 16 sites; however, it was found associated with *Ceratophyllum demersum* in two sites (St. 12 and 15), *Potamogeton perfoliatus* in two sites (St. 14 and 17), and with *Potamogeton crispus* in only one site (St. 25).

In summer, five submerged macropyte species representing four genera and four families (*Myriophyllum spicatum*, *Ceratophyllum demersum*, *Potamogeton perfoliatus*, *Potamogeton nodosus,* and *Najas marina*) were recorded. *Myriophyllum spicatum* was the most frequent species observed at 64.29% of the total sampling sites (Fig. 8). There were geographical differences in species distribution where *Myriophyllum spicatum* was found alone at 16 sites; however, it was associated with *Potamogeton perfoliatus* at St. 3, 6, 7, 13, and 19, *Ceratophyllum demersum* at St. 4, 5, and 6, and with *Potamogeton nodosus* at St. 3. *Ceratophyllum demersum* was found alone at St. 9; however, it was recorded as associated with *Potamogeton perfoliatus* at St. 6 and 12 and with *N. marina* at St. 10. *Potamogeton perfoliatus* was recorded alone at St. 25.

### Epiphytic diatoms

A total of 98 diatom species were recorded, consisting of 12 centric and 86 pennate species, belonging to 12 orders (Melosirales, Aulacoseirales, Thalassiosirales, Stephanodiscales, Fragillariales, Naviculales, Cymbellales, Thalassiophysales, Rhopalodiales, Bacillariales, Surirellales, and Achnanthales). In the winter, Stephanodiscales and Achnanthales were the most dominant, followed by Aulacoseirales and Fragilariales. From sites 1 to 10, *Cyclotella ocellata* was the dominant, followed by *Cocconeis placentula.* From sites 11 to 28, there is a fluctuation in dominance observed between the species *Cyclotella ocellata* and *Aulacoseira granulata*. *Cyclotella ocellata* recorded its highest percentage (67.37%) at St. 9, *Cocconeis placentula* (41.61%) at St. 4, and *Aulacoseira granulata* (50.11%) at St. 20. During the summer, Fragilariales, Achnanthales, and Stephanodiscales exhibited the highest level of dominance. *Fragilaria biceps* and *Cocconeis placentula* shared the dominancy from stations 1 to 6. However, *F. biceps* achieved its peak proportion of 58.86% of total diatoms at St 8. While *C. placentula* attained its highest relative abundance (44.50%) at St. 3.

**Fig. 9:**
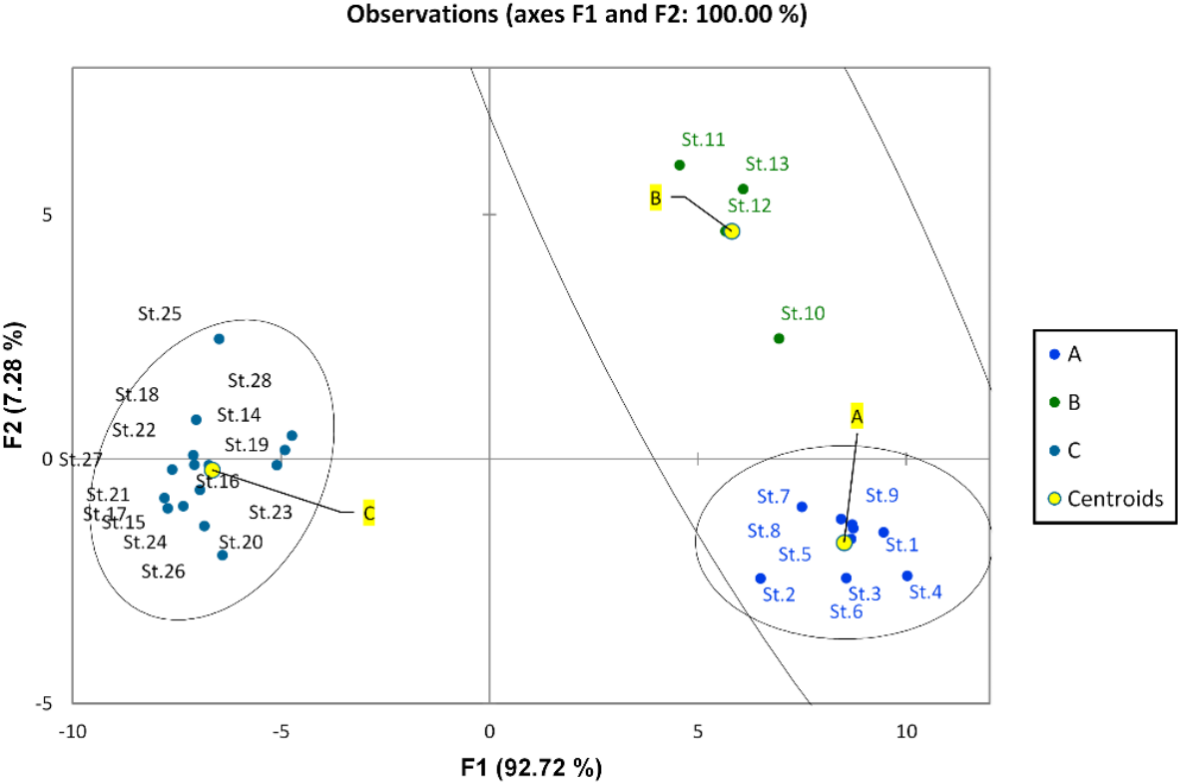
Discriminant Analysis clustered the Nile River sites into three groups (A, B and C) according to the distribution of epiphytic diatom species in 2022.

**Fig. 10:**
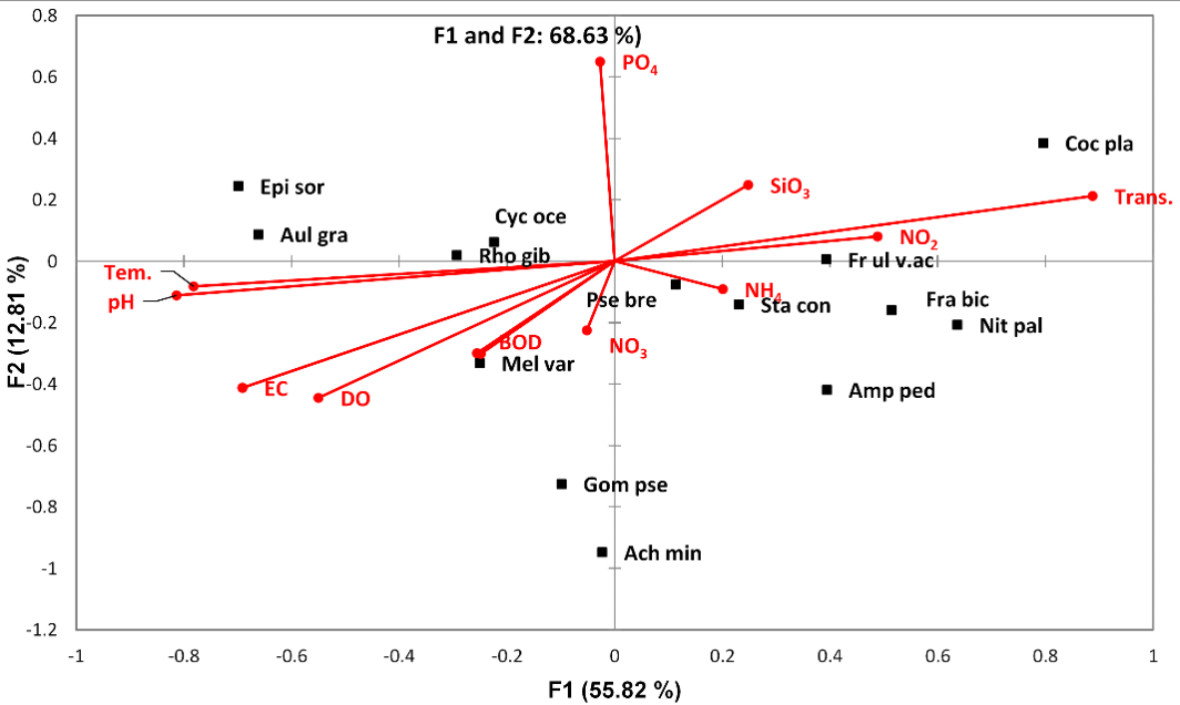
Canonical Correspondence Analysis (CCA) triplot diagram for the relations between the most common epiphytic diatoms species (squares) and the physicochemical variables (lines). The used parameters are abbreviated as follows: temperature (Tem,), transparency (Trans.), pH, electrical conductivity (EC), dissolved oxygen (DO), biological oxygen demand (BOD), chemical oxygen demand (COD), nitrite (NO_2_), nitrate (NO_3_), ammonia (NH_4_), phosphorous (PO_4_), silicate SiO_4_, The diatom species; *Epithemia sorex (Epi sor), Aulacoseira granulate(Aul gra), Cyclotella ocellata (Cyc oce), Rhopalodia gibba (Rho gib), Meloseira varians (Mel var), Pseudostaurosira brevistriata (Pse bre), Amphora pediculus (Amp ped), Nitzschia palea (Nit pal), Fragilaria ulna* var. *acus (Fr ul v. ac) and Cocconeis placentula* in the River Nile (2022).

The discriminant analysis divided the study area into 3 distinct groups: A, B, and C. Group A included sites from 1 to 9 stations; this group was dominated by *F. biceps*, *C. placentula,* and *Achanthidium minutissimum*. Group B was represented by stations 11, 12, and 13 and was predominately inhabited by *Amphora pediculus* and *Nitzschia palea*. Group C consisted of sites ranging from station 14 to 28 and was primarily characterized by the presence of *A. granulata*, *C. ocellata*, and *Epithemia sorex* (Fig. 9). According to the CCA analysis, environmental variables of the first two axes explained 68.63% of the variance in the weighted averages of diatom species. Transparency and dissolved oxygen are the crucial factors affecting the most dominant species, followed by pH, temperature, and PO_4_. *C. placentula, Nitzschia palea, and Amphora pediculus* correlated positively with transparency. *Epithemia sorex, Aulacoseira granulata,* and *Cyclotella ocellata* were affected positively by the presence of dissolved oxygen (Fig. 10).

### Epiphytic macroinvertebrates

**Fig. 11:**
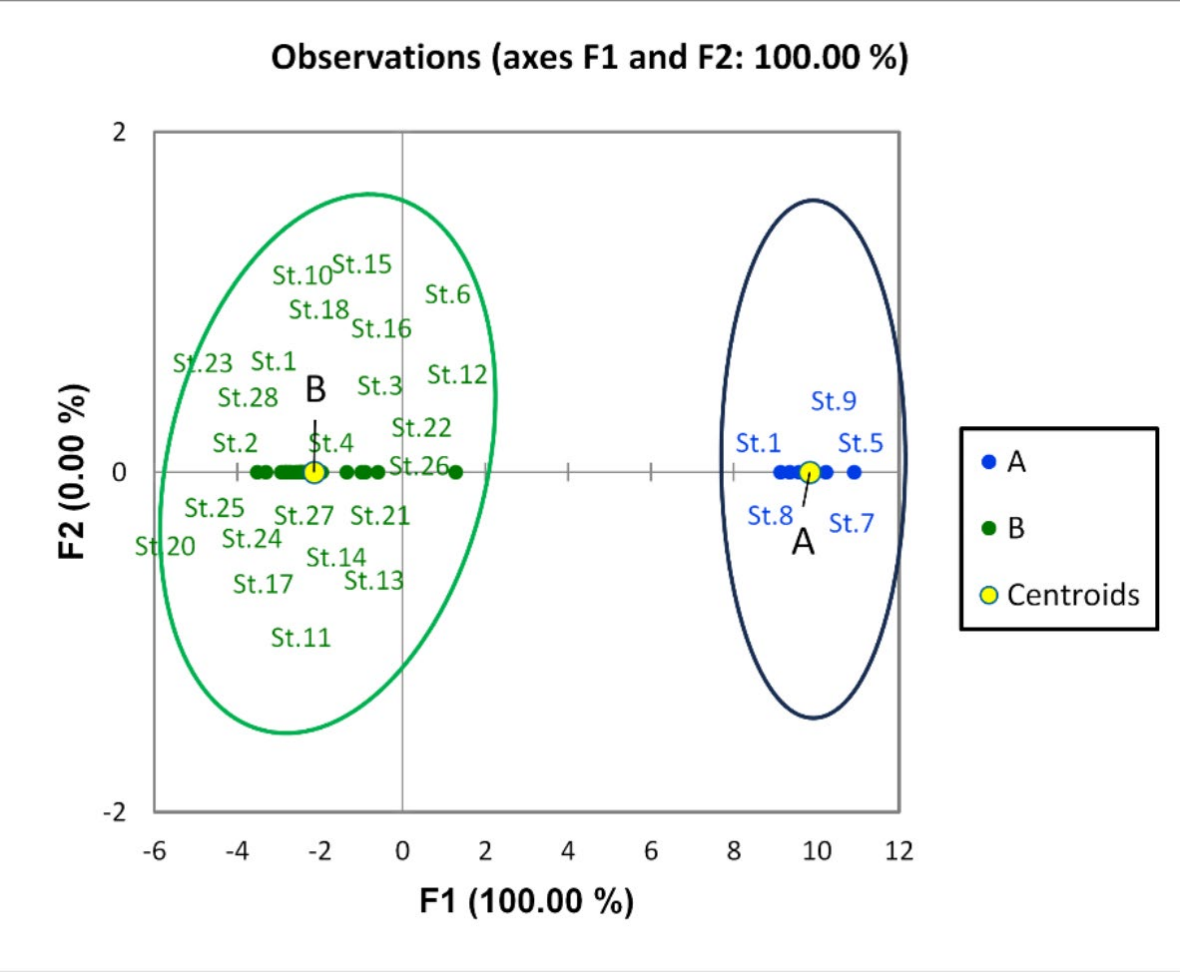
Discriminant analysis clustered the Nile River sites in two groups according to the distribution of epiphytic macroinvertebrates species in 2022.

**Fig. 12:**
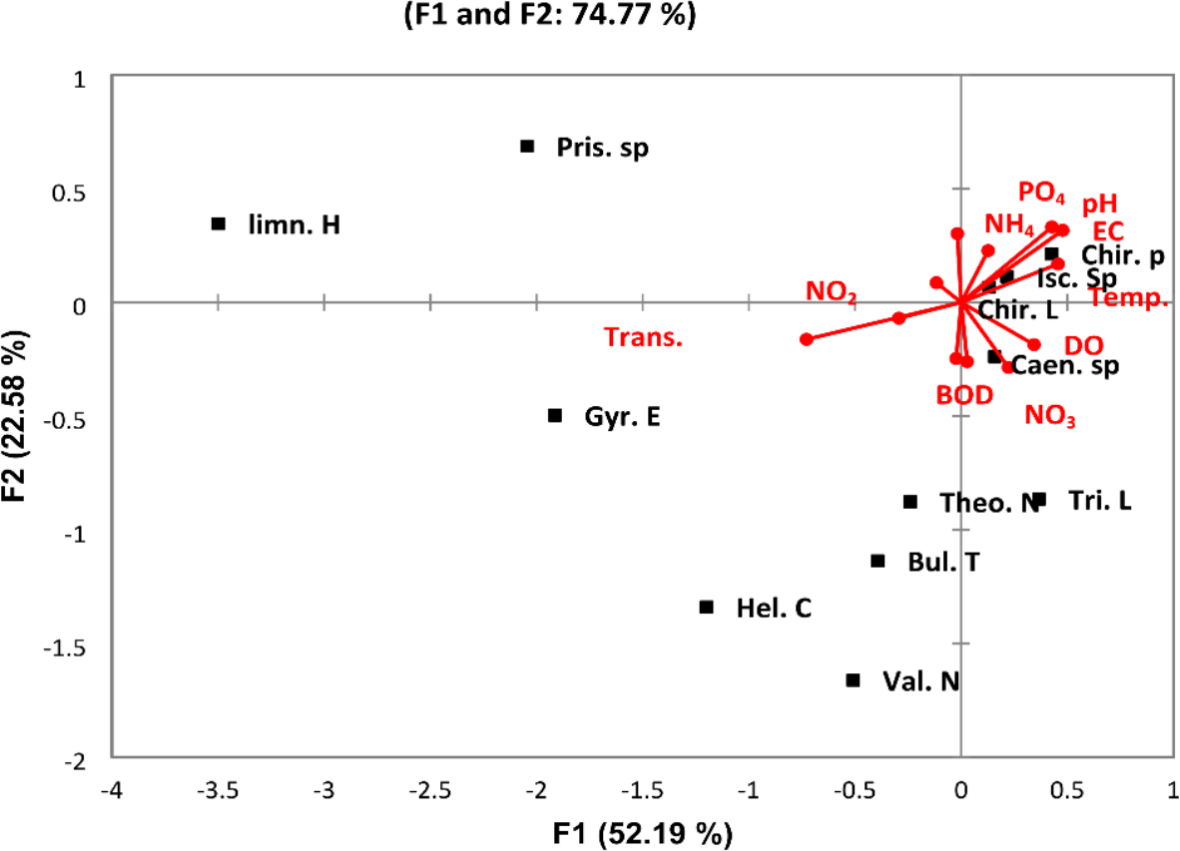
Canonical Correspondence Analysis (CCA) triplot diagram for the relations between the most common epiphytic macroinvertebrates species (squares) and the physicochemical variables (lines). The used parameters are abbreviated as follows: temperature (Tem,), transparency (Trans.), pH, electrical conductivity (EC), dissolved oxygen (DO), biological oxygen demand (BOD), chemical oxygen demand (COD), nitrite (NO_2_), nitrate (NO_3_), ammonia (NH_4_), phosphorous (PO_4_), silicate SiO_4_; Chironomid Larvae (*Chir. L*), *Chironomid* pupae (Chir. p), *limnodrilus hoffmeisteri* (limn. H), *Theodoxus niloticus* (Theo. N), *Gyraulus ehrenbergi* (Gyr. E), *Valvata nilotica* (Val. N), *Bulinus truncatus* (Bul. T), *Ischinura sp.* (Isc. Sp), *Caenis* sp (Caen. sp), *Helobdella conifera* (Hel. C), *Tricoptera Larvae (Tri. L)* and *Pristina* sp. (Pris. sp) in the River Nile (2022).

Macroinvertebrates associated with macrophytes in the River Nile from Aswan to Cairo belonged to four main phyla: Annelida constituted about 6.1% and was represented by 4 species; Arthropoda constituted about 83.4% and was represented by 13 species; Mollusca constituted about 9.8% and was represented by 12 species; and Hydrozoa constituted 0.5% and was only represented by one species. The average density of attached fauna in the whole area was 2877 organisms/kg. Arthropoda was the dominant group according to the number of species and density during this study. Chironomid larvae and pupae were the most dominant arthropods in the whole area. The discriminant analysis divided the sites into two groups (Fig. 11). Group A (the southern region) was represented by sites 1, 5, 7, 8, and 9 that have higher density and a higher number of species. Group B (most of them northern sites and a few southern sites) is represented by sites 2, 3, 4, 6, and sites from 10 to 28, where the density and number of species of macroinvertebrates attached to macrophytes decreased. Canonical Correspondence Analysis (CCA) was carried out to analyze ten environmental variables with 11 common species in the area investigated in Fig. 12. Analysis indicated that these factors do not have a strong influence on the distribution and abundance of species. The most important factors that affected this species were transparency, pH, and EC. The CCA biplot diagram showed that the transparency recorded a positive correlation with *Gyraulus ehernbergi*. There is a positive correlation between DO and *Caenis* sp., while DO have a negative correlation with *Limnodrilus* sp. and *Pistina* sp.

### Fish haematology

**Fig. 13:**
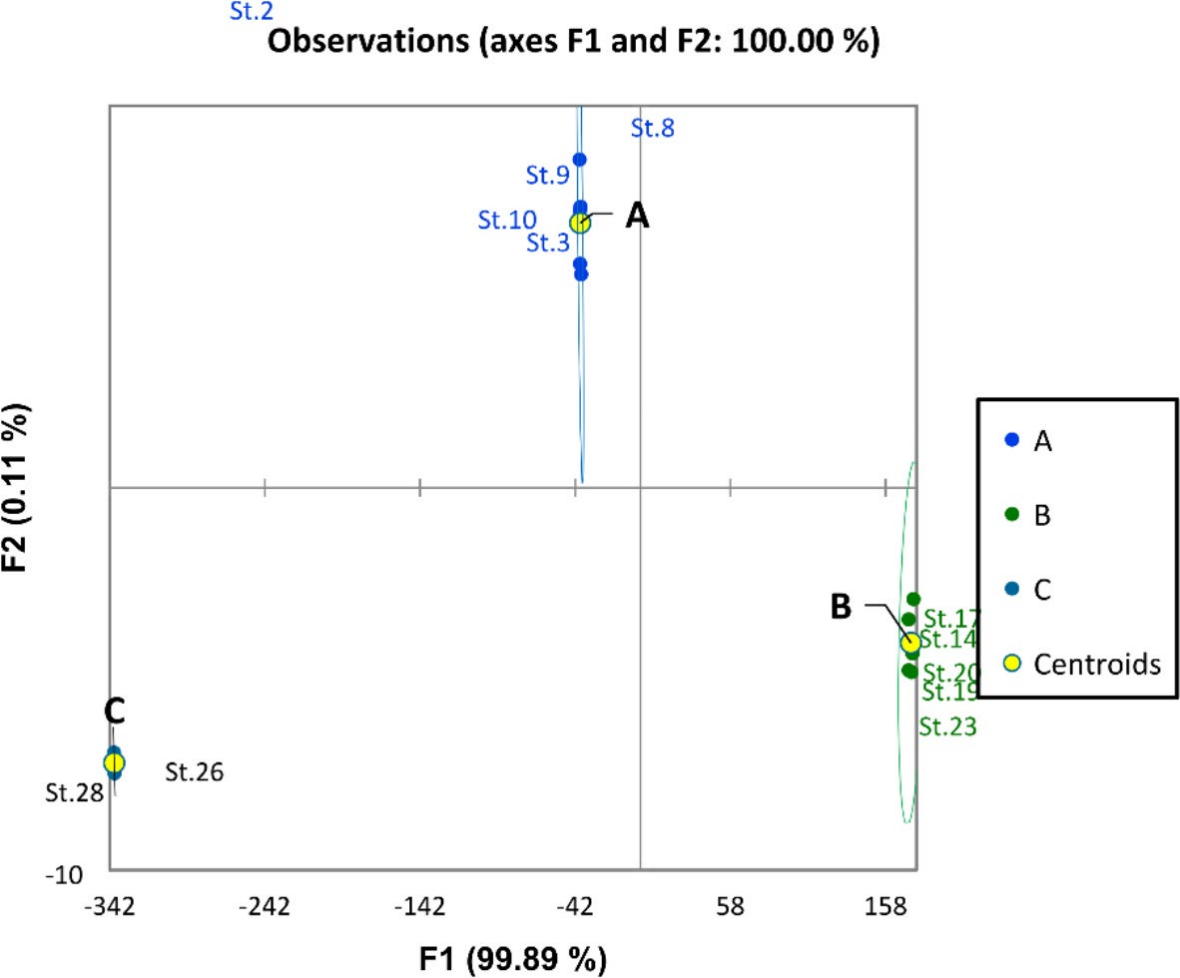
Discriminant analysis clustered the Nile River sites in two groups according to the results of fish hematology parameters in 2022.

**Fig. 14:**
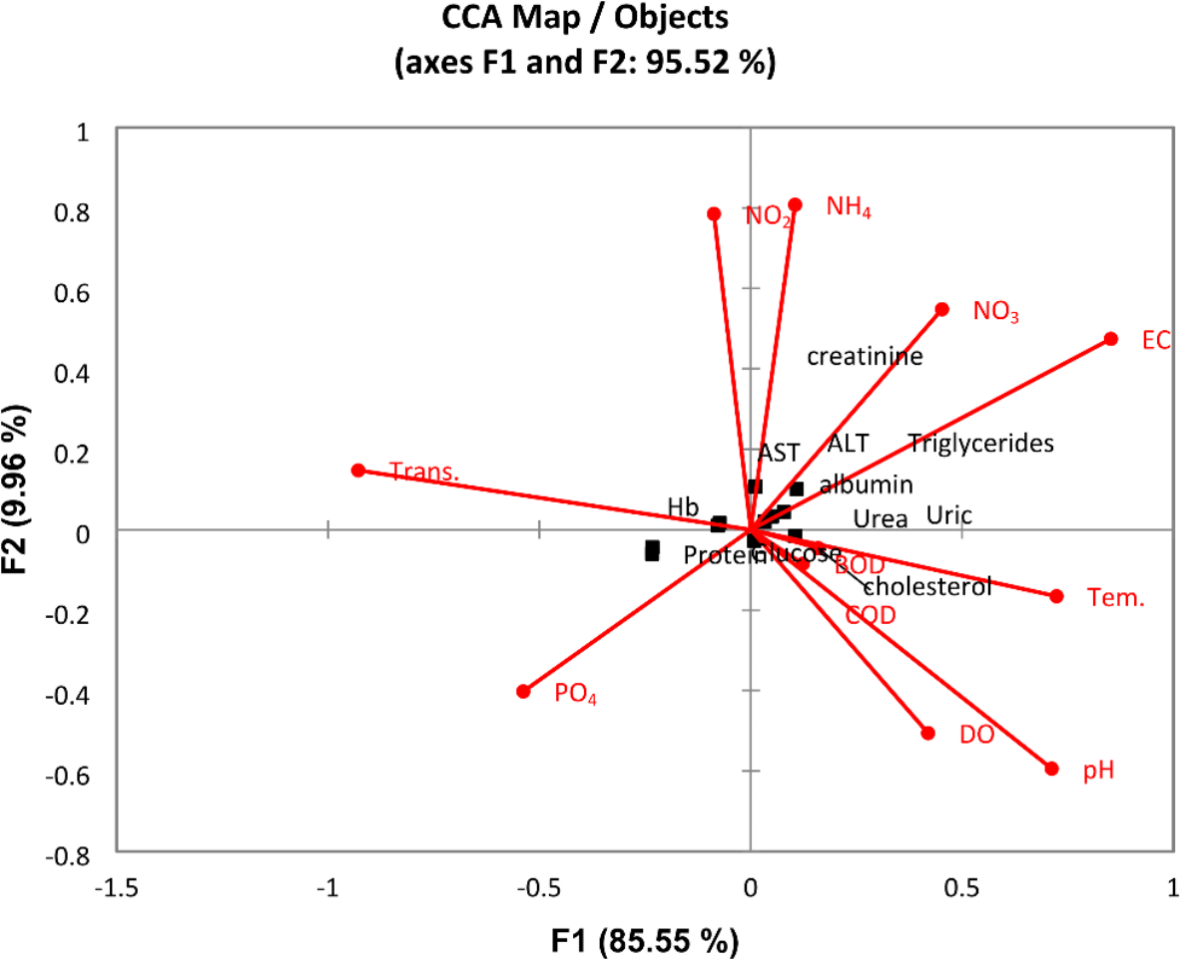
Canonical Correspondence Analysis (CCA) triplot diagram for the relations between the physicochemical variables (lines): temperature (Tem,), transparency (Trans.), pH, electrical conductivity (EC), dissolved oxygen (DO), biological oxygen demand (BOD), chemical oxygen demand (COD), nitrite (NO_2_), nitrate (NO_3_), ammonia (NH_4_), phosphorous (PO_4_), silicate SiO_4_, and the blood parameters (squares) of fish along River Nile (2022).

Based on the blood analysis results, all blood parameters ranged in narrow limits in all sites (governorates) except the Greater Cairo sites (sites 26 and 28). The results revealed that the blood parameters of the O. *niloticus* fish sample were within normal limits, especially during the summer, except that collected from Greater Cairo governorates stations. Analysis of the haematology parameters of the fish sampled revealed significant variations across the spatial habitats. Haemoglobin levels exhibited a clear trend of decreasing from south to north, with the highest values observed in Aswan (13.05 g/dL) and the lowest in Great Cairo (10.25 g/dL). Similarly, glucose levels demonstrated a northward increase, ranging from 134.36 mg/dL in Aswan to 178.11 mg/dL in Great Cairo. Conversely, total protein levels displayed a gradual decline from south to north, at recording the highest value (5.065 g/dL) at Aswan and the lowest value (3.17 g/dL) was recorded in the Great Cairo. Interestingly, albumin levels showed an opposite trend, increasing from 1.041 g/dL in Aswan to 2.048 g/dL in Great Cairo. lipid profile; triglyceride level were increased from Aswan (99.49 mg/dL) to Great Cairo (155.98 mg/dL). Cholesterol level followed a similar pattern, rising from 93.855 mg/dL in Aswan to 182.5 mg/dL in Great Cairo. Notably, urea, uric acid, and creatinine levels also demonstrated a north–south gradient, with higher concentrations observed in the northern sites. This suggests potential differences in metabolic activity and the overall health status of fish populations inhabiting different regions of the Nile River. The discriminant analysis divided the sites into three groups based on the different concentrations of blood parameters (Fig. 13). Where group A (the southern region) was represented by sites 2, 3, 8, 9, and 10. This group was characterized by the lower values of all blood parameters except Hb and total protein. Group B (the middle and some sites of the northern regions) was represented by sites 14, 17, 19, 20, and 23. This group was characterized by the medium values of all blood parameters. Group C, included sites 26 and 28 (Greater Cairo), was characterized by the higher values of Hb and total protein and the lower values of other blood parameters. The CCA analysis showed that Hb and T. protein were more affected by transparency and PO_4_, while the other blood parameters were affected by other water quality parameters (Fig. 14).

## Discussion

Assessing the quality of Nile River water is very important to know the practicality of using Nile water as a source for various activities, such as its relevance for aquatic life, irrigation, and drinking^6,7^. Water quality is evaluated using physical, chemical, and biological properties that indicate the characteristics of water and variables that affect water quality in relation to the presence of life, especially human activity. It is predetermined by its intended uses, and each of these uses affects it^49^. The temperature in the current study falls within the range that is best for fish and aquatic organisms in the winter and summer seasons, with a highly significant difference between the two evaluated seasons. Temperature is negatively correlated with pH and DO, which may be attributed to the increase in temperature decreasing the solubility of gases in water such as CO_2_ and DO. As well, it has a clear effect on the photosynthesis rate of phytoplankton^2,50^. The transparency of the water signifies the depth of the photic zone^51^, it gradually decreased from south to north, which may be attributed to the intrusion of many wastes to Nile River. This observation imitated by the strong negative correlation of the transparency with EC, TDS, TSS. That increased from south to north. These findings agree with those obtained by Goher et al.^13^ and El Sayed et al.^9^. They concluded that the transparency values were affected by the particulate content in the water from suspended matter and floating substances and is related to human activities downstream of the Nile River^13^. The pH of the Nile lies on the alkaline side, with highly significant differences among sites. The pH value increased gradually from south to north and recorded the lowest pH value (7.41) in summer at St. 1 downstream Aswan Reservoir, which is combined with lower activities of the phytoplankton. This finding may be due to the high velocity of water current. Also, low pH value (7.64) was detected in site St. 2 (after Kima Drain) in winter seasons, which may be attributed to the discharge of liquid waste that is heavily laden with organic and inorganic components. These results were established by the negative correlation of pH with NH_4_ (Fig. 3), while the pH values were positively correlated with the DO, which definite the effect of photosynthesis on raising the pH value^6^. DO is an important parameter in assessing pollution levels in a particular water body^52^. The DO results show a narrow variation, with decreased in the southern stations, especially at sites 1, 2, and 3 during summer due to the temperature raising, which decreases the solubility of DO^1,50^. These results are proven by the high negative correlation between DO and temperature (Fig. 3). Our results show that the levels of BOD and COD were within the internationally permissible levels and showed a narrow fluctuation between season and sites. This may be due to the dilution effect and self-purification of the Nile water. These results are less than those recorded in 2011^6^ and 2017^7^. The Nile water, in general, contains low concentrations from NO_2_, NO_3_, NH_4_, PO_4_, and SiO_4_, and fluctuated in a narrow range from site to site along the Nile; these values were lower than those recorded by Hussein et al.^7^, Abdel Sater et al.^6^ and Abdel Satar^11^. On the other hand, the recorded value falls within the permissible limits for all uses.

In general, the physicochemical parameters in River Nile water were within the acceptable criteria for drinking water according to the Egyptian drinking water quality standards^53^, for irrigation water according to the FAO^54^, and for the life of aquatic organisms according to standards of the CCME^55^, which reflects the good quality of the Nile water and its suitability for different purposes. This may be attributed to the dilution effect due to the increase in water level, self-purification, and treatment of several drain’s wastewater discharged into the Nile body. This result is in agreement with the result obtained by Abdel-Shafy and Aly^56^, who pointed out that the Nile water quality showed some improvement over average during the high flood period, where the excess water behind the AHD is drained into the Nile River, replenishing the water in the river. Abdel-Satar^11^ concluded that the water quality of the mid-river Nile remains at an average clean level due to the dilution effect and degradation of discharged pollutants. El Sayed et al.^50^ explained that the water quality of the Ismailia Canal is excellent for various uses because of the high dilution effect in last years, which renews the water in the canal and decomposes the pollutants that are discharged by self-purification, which led to the reduction of pollutants. On the other hand, this result is in disagreement with the result obtained by Hussein et al.^7^, who reported that a total of 35% of the samples were classified as ‘fair’ for drinking water resources. Abdel Satar et al.^6^ validate that the river became unfit for aquatic life, whereas the WQI of aquatic life indicated that the Nile water quality deteriorated and extended from poor to margin. Ali et al.^5 ^reported that the River Nile water at Aswan governorate is heavily polluted, especially at El-Sail Drain and Kom Ombo Drain. This can be attributed to the decrease in the amount of water discharged into the Nile River from Lake Nasser during this period and the effect of the sample location close to the pollution sources. Discriminant analysis revealed spatial variation in water quality based on transparency value, EC, TDS, pH, DO, BOD, and COD that grouped the Nile River into 3 groups in the south, middle, and north. This can be mainly attributed to the high distance of the study area with many drains entering along the river Nile^57,58^.

Many studies have shown that the phytoplankton composition and diversity are closely related to physicochemical factors^2,30,59–61^. The spatial fluctuations and succession in phytoplankton assemblages in the Nile River are caused by the effects of pH, water temperature, nutrient concentrations (nitrogen and phosphorous), human activities, discharge of effluents, and hydrodynamic conditions. The increase in the phytoplankton densities and species richness in the Nile water downstream is consistent with previous studies on the Nile water^62–65^. The dominant species in the Nile River were not significantly impacted by time. In contrast, the abundant species in the present study are like those in other studies on the Nile water and are known as good survivals for a wide range of pollutant sources^62,63,66^. The low recorded phytoplankton densities in the southern stations from St. 2 to St.12 are associated with the low nutrient content in these sites, and the dominance of *Synechococcus* sp. and *Navicula muralis* in these stations affected by the industrial effluent was noted formally by Abdel-Aal^17^ and Abdel-Hamid et al.^18^.

Zooplankton showed high variation in their distribution, abundance, and diversity along the Nile River. Therefore, the discriminant analysis divided the river sites into three groups based on zooplankton data. The first group in south represented from St. 1 to St. 11. This group is characterized by the lowest density and diversity of zooplankton. The low density and diversity of zooplankton in these sites is associated with low nutrients and phytoplankton, as mentioned previously in the chemical and phytoplankton sections. Thus, these results indicated low eutrophication in these sites. Nevertheless, zooplankton was highly abundant and diverse in the third group (Great Cairo), which includes from St. 21 to St. 28 (downstream). This result is associated with the high content of nutrients and phytoplankton and indicates a high eutrophication level in these sites. Furthermore, these sites were characterized by a high abundance of trophic indicator species such as *Keratella cochlearis, Brachionus calcyflouris, Philodina roseola, Polyarthra vulgaris, Collotheca ornate, Bosmina longirostris, Chydorus sphaericus,* and *Vorticella campanula.* Yağci^67^ mentioned that the high abundance of *B. calyciflorus*, *K. cochlearis,* and *P. vulgaris* is an indicator of the high eutrophication. Hegab and Khalifa^68^ recorded the highest abundance of *B. calyciflorus*, *P. roseola*, and *K. cochlearis* in the highly eutrophicated and polluted sites in the Rosetta Branch of the Nile. Bhandarkar^69^ also pointed out that the genus Brachionus rapidly increases under high eutrophication conditions. On the other hand, the high abundance and absolute dominance of rotifers in the northern part of the Nile gives evidence of a trophic status in which phytoplankton abundance is reliably linked to zooplankton abundance^70^.

The diversity and abundance of aquatic macrophytes are extensively affected by changes in environmental factors, including water physicochemical characteristics, competitive interactions between different organisms, and sediment texture^71^. Compared with the previously recorded results, a total of 21 submerged macrophyte species, dominated by 8 species, were recorded in the Nile as a whole by Zahran^72^. However, Hussian and Haroon^73^ recorded *Myriophyllum spicatum*, *Ceratophyllum demersum,* and *Potamogeton perfoliatus* as the only submerged macrophytes at the Nile River with the highest presence percentage for *Myriophyllum spicatum* (80%). This variation in species numbers could be related to the variation in sampling sites, environmental conditions, as well as the effect of human impact on water bodies. The occurrence of aquatic macrophytes is unambiguously related to water chemistry, and using plant species or communities as indicators or biomonitors is an objective for surveying water quality^74^. During this study, the recorded species indicated that northern sites are rich in nitrites, nitrates, and phosphates. However, the indicator species of highly polluted water with organic matter and industrial wastes (such as *Potamogeton pectinatus*) were not detected in the study area.

A total of 98 epiphytic diatom species, 12 centric, and 86 pennate species belonging to 12 orders were identified. The discriminant analysis divided the sites into 3 groups: A, B, and C. Groups A and C are the main two groups. It may be a discriminant analysis of clustered stations depending on regions where close sites were clustered together or according to indicator species. Group A includes sites from 1 to 9 (southern part). These sites were characterized by low nutrients, as mentioned in the chemistry section. The sites of group A gathered due to the presence of indicator species *Fragilaria biceps*, Cocconeis *placentula,* and *Achanthidium minutissimum. Cocconeis placentula and was* correlated positively with transparency and Nitrite. *Achnanthedium minutissimum* has the highest weighted average for DO and BOD and the lowest weighted average for PO_4_ and SiO_4_. *Fragilaria biceps* and *Cocconeis placentula* are often found in mesotrophic to eutrophic waters, and *Achnanthidium minutissimum* is found in well-oxygenated, clean, fresh waters^41,75^. So, the mentioned species are indicators of clean sites. Group B (sites. 10, 11, 12, and 13) with indicator species of *Nitzschia palea* and *Amphora pediculus* that correlated positively with nitrate and ammonium, in agreement with the study of Arumugham et al.^76^. Group C includes stations from 14 to 28, which represented the five northern governorates. These sites contain high nutrients and include indicator species: *Aulacoseira granulata*, *Cyclotella ocellata, and Epithemia sorex. Aulacoseira granulata* that prefers warm temperature. *Epithemia sorex* was linked positively with pH, PO_4_, and SiO_4_. *Epithemia sorex* is a cosmopolitan species found in both flowing and standing waters of moderate to high electrolyte content. Also extending into brackish biotopes, *Cyclotella ocellata* occurs in meso- to eutrophic waters with an elevated pH^41^. Several workers reported that some dominant species indicate the water quality. For instance Eminsoon and Moss^77^ and Marker and Collet^88^ explained that *Cocconeis placentula* grows well under a wide range of conditions, and *Niztschia amphibia*, *Niztschia palea,* and *Gomphonema parvulum* are generally recognized to be indicators of polluted waters. They also concluded that *Achnanthidium minutissimum* and *Fragilaria ulna* are generally found in less polluted but eutrophic waters. Site 22 contains *Anomoeneis sphaerophora*, *Gomphonema parvulum f. saprophilum,* and *Encyonema caespitosum,* which are tolerating critical levels of pollution. This means that the downstream sites in the future are supposed to be polluted, but the upstream stations from 1 to 9 have good-quality water.

For attached macroinvertebrates, Arthropoda was the dominant group according to the number of species and density during this study; this agrees with Khalil et al.^79^ in the Nile River from Aswan to Cairo. Insects were the dominant group attached to macrophytes, and Chironomid larvae were the most dominant insect. This result agrees with the results of El-Damhogy et al.^80^, who stated that macroinvertebrates associated with the submerged plants are rich in insects in the Nile River from Aswan to Cairo and confirmed that chironomid larvae have the highest population density. Chironomidae can act as an indicator of pollution. Bendary and Ibrahim^81^ also confirmed this result at El Rayahs El-Behery and El-Nassery. From the data analysis, *Caenis* sp. is closely associated with DO. This agrees with the findings of Goulart and Callisto^82^, who found that Ephemeroptera species are well-represented in high-quality environments because they require high concentrations of dissolved oxygen in water. The presence of some species as Tricoptera larvae and some species of Mollusca as *Gyraulus* sp. indicated that most selected sites in the investigated area were not polluted. DO has a negative correlation with *Limnodrilus* sp. and *Pistina* sp. This may be due to their tolerance to the low oxygen content in anoxic conditions of the bottom sediment^83,84^. The diversity and density of macrobenthic fauna associated with macrophytes were lower at some sites, as in station 2 near kema factories and a few other stations with organic pollution from some drains. These results agree with Abdel Gawad^85^, who stated that the water quality in the River Nile from Aswan to Cairo was good except a few stations in front of some drains and factories that were heavily polluted. The abundance of macroinvertebrates attached to macrophytes is affected by many factors other than physicochemical parameters, such as the type of macrophyte, the structure of leaves, and the larger surface of leaves. It is also affected by predators, water turbulence, and wave intensity.

Blood laboratory results of fish samples showed that they were within normal limits in all studied sites due to the improvement of water quality in the Nile River, except et al.-Qanater Al-Khayriyah and El-Tabbin in Greater Cairo, where hemoglobin and total protein values decreased in these sites. This may be due to exposure to various stressors in this area^86^. This result supports the hypothesis of Moharam et al.^87^, who reported an adaptive response of fish exposed to pollution or stress by impairing hemoglobin synthesis, ultimately leading to a state of hypochromic anemia, macrocytic anemia, and erythrocyte swelling. Also, a decrease in total plasma proteins may be the result of exposure to heavy metals. They attributed this decrease to kidney excretion, poor protein synthesis, or liver disorder. On the other hand, this decrease can result from the degradation of protein into amino acids and then into nitrogen and other elementary molecules^88^. Also, the results of glucose, albumin, triglycerides, cholesterol, urea, uric acid, and creatinine were increased in El-Qanater El-Khyria. This may be due to the decrease of water quality and increase of pollutants in this area, especially in the winter, as a result of the relative decrease in water levels in this season. High values of these parameters have been previously reported in fish exposed to different pollutants^89–93^.

## Conclusion

This comprehensive study of the Nile River water quality from Aswan to Cairo reveals the pattern of changes in water quality along the Nile River in Egypt based on chemical and biological factors. The study focused on collecting samples from different sites that represent the social and behavioral changes of the different governorates along the Nile. The sampling process in this study did not focus on drain outlet sites as in previous studies but rather represented each governorate with several sites based on distances to make a definitive judgment on the water quality in general and not just the locations of pollution. The results showed that the southern regions have higher transparency and lower levels of nutrients and eutrophication indicators, indicating a less affected environment. In contrast, the northern sites, especially around Greater Cairo, show lower transparency, higher concentrations of eutrophication indicators, and nutrients to indicate the high levels of eutrophication. This gradient is likely driven by increased human activities, agricultural runoff, and industrial waste into the river. These results were confirmed by various biological studies of phytoplankton, algae, zooplankton, and invertebrates, as well as fish blood characteristics. The indicator species of pollution were absent in the southern part, while they appeared somewhat high in the north. Our results show a gradual improvement in the quality of the Nile water compared to previous studies, possibly due to increased water levels and the treatment of some wastewater discharges. However, the northern sites still have high levels of chemical and biological trophic indicators. Therefore, this area needs continued efforts to manage pollution sources and ensure the long-term health of the Nile River in Great Cairo.

## Electronic supplementary material

Below is the link to the electronic supplementary material.

Supplementary Material 1

## Data Availability

All data generated or analyzed during this study are included in this published article [and its supplementary information files].
