# Supplementary material for: Evaluating the spatial pattern of water quality of the Nile River, Egypt, through multivariate analysis of chemical and biological indicators
Source: Sci Rep. 2025 Mar 4;15:7626. doi: 10.1038/s41598-025-89982-2 (PMC11880377; doi:10.1038/s41598-025-89982-2)
Supplement: Supplementary file 1 — Supplementary Material 1 [file 41598_2025_89982_MOESM1_ESM.docx]

**Supplementary File**

.

| **Province** | **Site** |  | **Latitude (N)** | **Longitude (E)** |
| --- | --- | --- | --- | --- |
| **Aswan** | 1 |  | 24.042195 | 32.86433 |
|  | 2 |  | 24.130773 | 32.89028 |
|  | 3 |  | 24.460985 | 32.91381 |
|  | 4 |  | 24.813555 | 32.90834 |
|  | 5 |  | 25.014504 | 32.8778 |
| **Luxor** | 6 |  | 25.450289 | 32.53505 |
|  | 7 |  | 25.618721 | 32.57438 |
|  | 8 |  | 25.710258 | 32.64254 |
| **Qena** | 9 |  | 25.930148 | 32.75422 |
|  | 10 |  | 26.14981 | 32.70213 |
|  | 11 |  | 26.115375 | 32.46312 |
|  | 12 |  | 26.132135 | 32.17651 |
| **Sohag** | 13 |  | 26.385482 | 31.86447 |
|  | 14 |  | 26.592076 | 31.7102 |
|  | 15 |  | 26.763459 | 31.55485 |
| **Asyut** | 16 |  | 27.104496 | 31.31901 |
|  | 17 |  | 27.188309 | 31.19483 |
|  | 18 |  | 27.439194 | 30.85972 |
| **Menyia** | 19 |  | 27.985369 | 30.8543 |
|  | 20 |  | 28.086865 | 30.7698 |
|  | 21 |  | 28.484527 | 30.83475 |
| **Beni Suef** | 22 |  | 28.811871 | 30.90749 |
|  | 23 |  | 29.05808 | 31.10115 |
|  | 24 |  | 29.348297 | 31.2147 |
| **Great Cairo** | 25 |  | 29.573823 | 31.26996 |
|  | 26 |  | 29.775975 | 31.28995 |
|  | 27 |  | 29.960117 | 31.24291 |
|  | 28 |  | 30.152418 | 31.15795 |

**Table S1.** Coordinates of the sampling locations.
